# Variants of *BEST1* and *CRYBB2* cause a complex ocular phenotype comprising microphthalmia, microcornea, cataract, and vitelliform macular dystrophy: case report

**DOI:** 10.1186/s12886-023-02915-3

**Published:** 2023-04-19

**Authors:** Jie Shi, Tengyang Sun, Ke Xu, Xin Zhang, Yang Li

**Affiliations:** Beijing Institute of Ophthalmology, Beijing Tongren Eye Center, Beijing Tongren Hospital, Capital Medical University, Beijing Ophthalmology and Visual Sciences Key Lab, Hougou Lane 17, Chong Nei Street, Beijing, 100730 China

**Keywords:** Best vitelliform macular dystrophy, Cataract, *BEST1* gene, *CRYBB2* gene, Case report

## Abstract

**Background:**

Best vitelliform macular dystrophy (BVMD), caused by pathogenic variants of the *BEST1* gene, has not been reported in association with cataracts and ocular malformations. We reported a case with a complex ocular phenotype comprising microphthalmia, microcornea, cataract, and vitelliform macular dystrophy.

**Case presentation:**

A six-year-old girl manifested photophobia and a poor visual behavior. A thorough ophthalmic examination revealed the patient to have bilateral microphthalmia, microcornea, congenital cataract, and Best vitelliform macular dystrophy (BVMD). Whole exome sequencing (WES) identified one variant in the *BEST1* and one variant in *CRYBB2* genes: c.218 T > G p.(Ile73Arg) and c.479G > C p.(Arg160Pro). The first variant was inherited from the proband’s father, who was diagnosed with subclinical BVMD, while the second was a de novo variant. A minigene assay showed that c.218 T > G in *BEST1* did not affect pre-mRNA splicing.

**Conclusions:**

This case suggests that the complex ocular phenotype comprising BVMD and congenital cataract with microphthalmia cannot be explained by variation in one gene but is caused by variants in *BEST1* and *CRYBB2*. This case highlights the importance of general clinical evaluation and comprehensive genetic testing for diagnosing complex eye diseases.

**Supplementary Information:**

The online version contains supplementary material available at 10.1186/s12886-023-02915-3.

## Background

Best vitelliform macular dystrophy (BVMD, OMIM 153700) is an inherited macular degeneration caused by pathogenic variants of the *BEST1*gene (OMIM 607854). This gene encodes bestrophin-1, an integral transmembrane protein [1], and its variants can result in at least five clinically distinct retinal degenerative diseases: BVMD, adult-onset vitelliform macular dystrophy (OMIM 153700), autosomal dominant vitreoretinochoroidopathy (ADVIRC, OMIM 193220), retinitis pigmentosa (OMIM 613194), and autosomal recessive bestrophinopathy (OMIM 611809) [1–5]. These phenotypes are collectively known as “bestrophinopathies” and involve the entire eye from anterior to posterior segments: microcornea, short axial length, glaucoma, early onset cataract, and retinal degeneration.

Congenital cataract (CC) is a leading cause of childhood blindness. About one-third of the cases are hereditary [6]. At present, over 60 genes have been identified that cause CC, ranging from crystallin, connexin, lens membrane, and cytoskeleton-related genes to transcription factors and other functionally divergent genes (https://cat-map.wustl.edu/, August 25, 2022). The crystallins account for up to 90% of the water-soluble proteins in the lens, and variants in crystallin genes are most common in CC [7]. Previous studies have reported that some variants in crystallin genes (e.g., *CRYBB1*, *CRYBB2*, *CRYAA*, and *CRYGC*) could lead to a combined occurrence of CC and other ocular abnormalities, such as microcornea, microphthalmia, coloboma, and glaucoma [6, 8].

Here, we report a patient suffering from microphthalmia, microcornea, CC (OMIM 601547), and BVMD caused by novel heterozygous variants in *BEST1* and *CRYBB2* (OMIM 123620). Our findings expand the variant spectrum of *CRYBB2* and *BEST1* and provide novel insights into the molecular and clinical analyses of complex inherited ocular disease phenotypes.

## Case presentation

**Fig.1:**
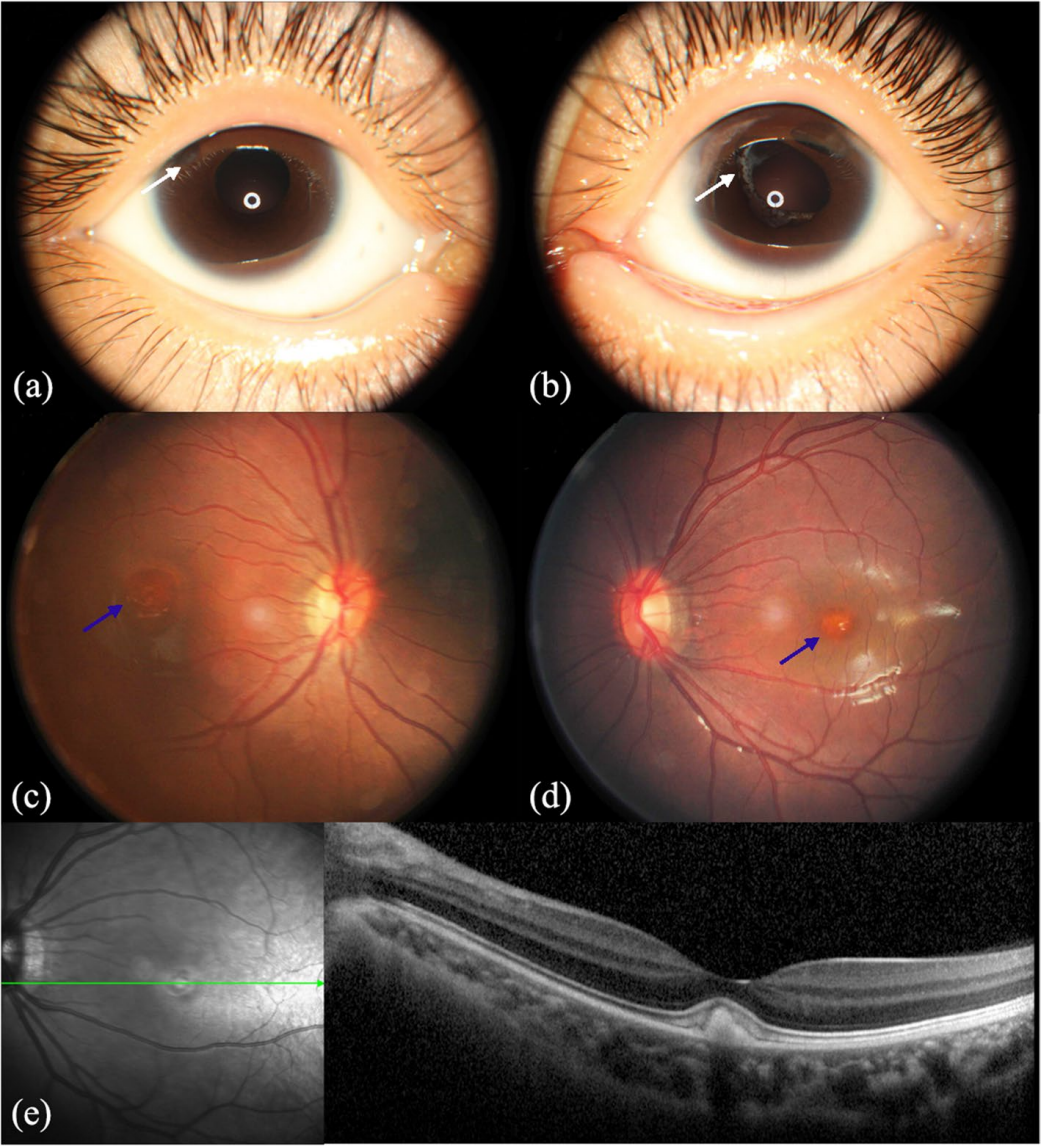
Ophthalmic examinations of the proband. **a**,**b** Anterior segment photographs show opaque capsular remnants (white arrow) in the pupillary area or peripheral iridotomy in aphakic eyes. **c**,**d** Fundus photographs show vitelliform lesions (blue arrow) in both eyes. **e** Optic coherence tomography (OCT) images show subretinal detachment and deposits of hyperreflective material located at the RPE level

A six-year-old girl had manifested photophobia and a poor visual behavior since 6 months of age and was diagnosed with congenital cataract, microcornea, and microphthalmia. She had undergone bilateral phacoemulsification, peripheral iridotomy, and anterior vitrectomy in our hospital when she was 1.5 years old. She had elevated intraocular pressure (IOP) of 29 mmHg (OD) and 24 mmHg (OS) at age 4 and was treated with carteolol and travoprost eye drops. The patient did not show any other systemic abnormalities. No family history of similar disease was reported. Her best corrected visual acuity (BCVA) at the last examination was 20/100 (OD) and 20/60 (OS). The spherical equivalent refraction was + 12.5 diopters and + 12 diopters in the right and left eyes, respectively. The IOP was normal. She had nystagmus and bilateral small vertical/horizontal palpebral aperture. The horizontal cornea diameter was 6 mm in both eyes, and the axial length was 17.7 mm (OD) and 18.9 mm (OS). A slit lamp examination demonstrated a round but upwardly displaced pupil in the right eye and a deformed pupil and peripheral anterior synechia in the left eye. In addition, opaque capsular remnants in the periphery of the pupillary and peripheral iridotomy apertures were observed in both aphakic eyes (Fig. 1a and b). A fundus examination showed a bilateral normal cup-to-disc ratio of 0.4 and yellowish egg-yolk lesions in the macular region (Fig. 1c and d). Spectral-domain optical coherence tomography (SD-OCT) revealed bilateral subretinal deposit of hyperreflective material in the fovea (Fig. 1e). Fundus autofluorescence (FAF) images and electrooculogram (EOG) could not be acquired owing to the proband’s young age.

**Fig. 2:**
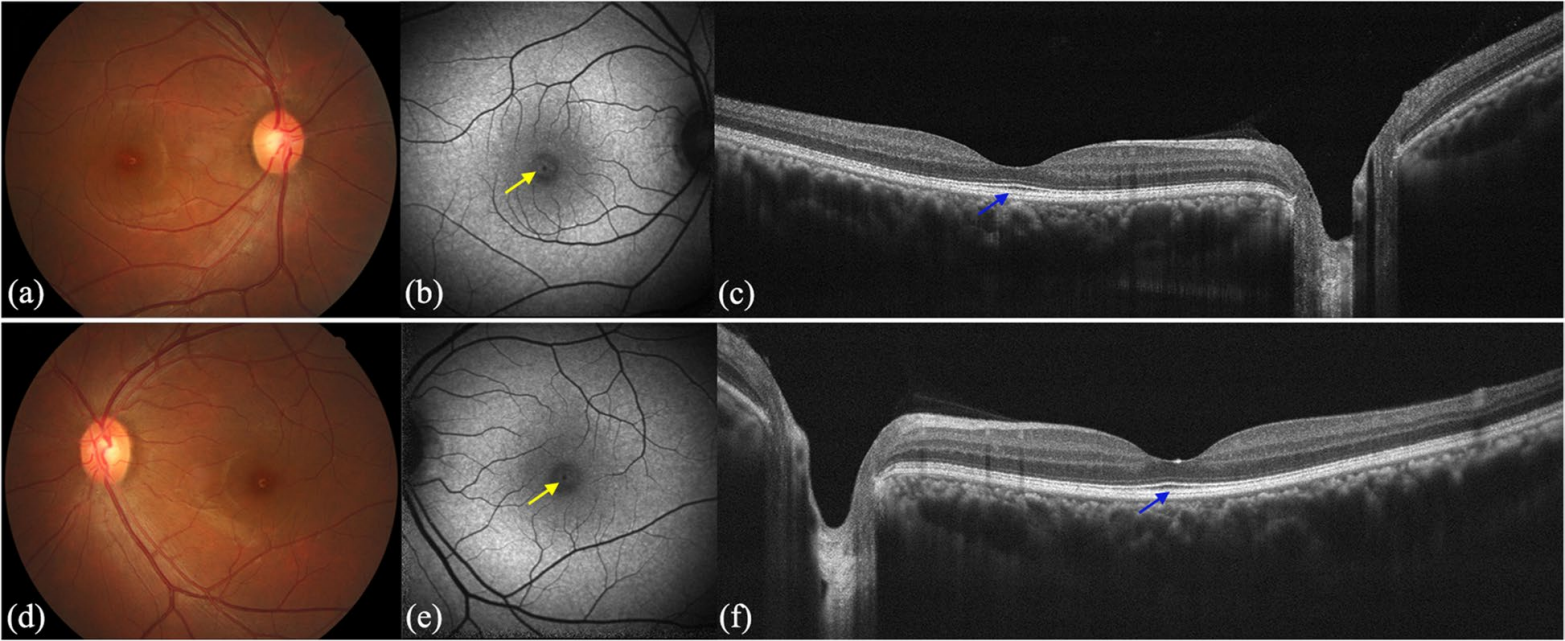
Ophthalmic examinations of the proband’s father (I:1). **a**,**d** Fundus photographs show hypo-pigmented changes in the fovea. **b**,**e** FAF images show a narrow ring of increased autofluorescence (yellow arrow) in the fovea of both eyes. **c**,**f** OCT images present a thicker and more reflective appearance of the interdigitation zone (blue arrow) in both eyes

The proband's father had no symptoms. The BCVAs were 20/20 in both eyes. His slit lamp examination was unremarkable, and his horizontal cornea diameter was 12 mm in both eyes. Fundoscopy revealed a hypopigmented change in the fovea of both eyes (Fig. 2a and d). FAF demonstrated a narrow ring of increased autofluorescence in the fovea (Fig. 2b and e). The OCT images showed a bilaterally thicker and more reflective interdigitation zone in the macula (Fig. 2c and f). The EOG examination showed Arden ratios of 1.1 in both eyes (normal value: 1.8–3.0). The ophthalmic examinations of the proband's mother and sister were normal.

**Fig.3:**
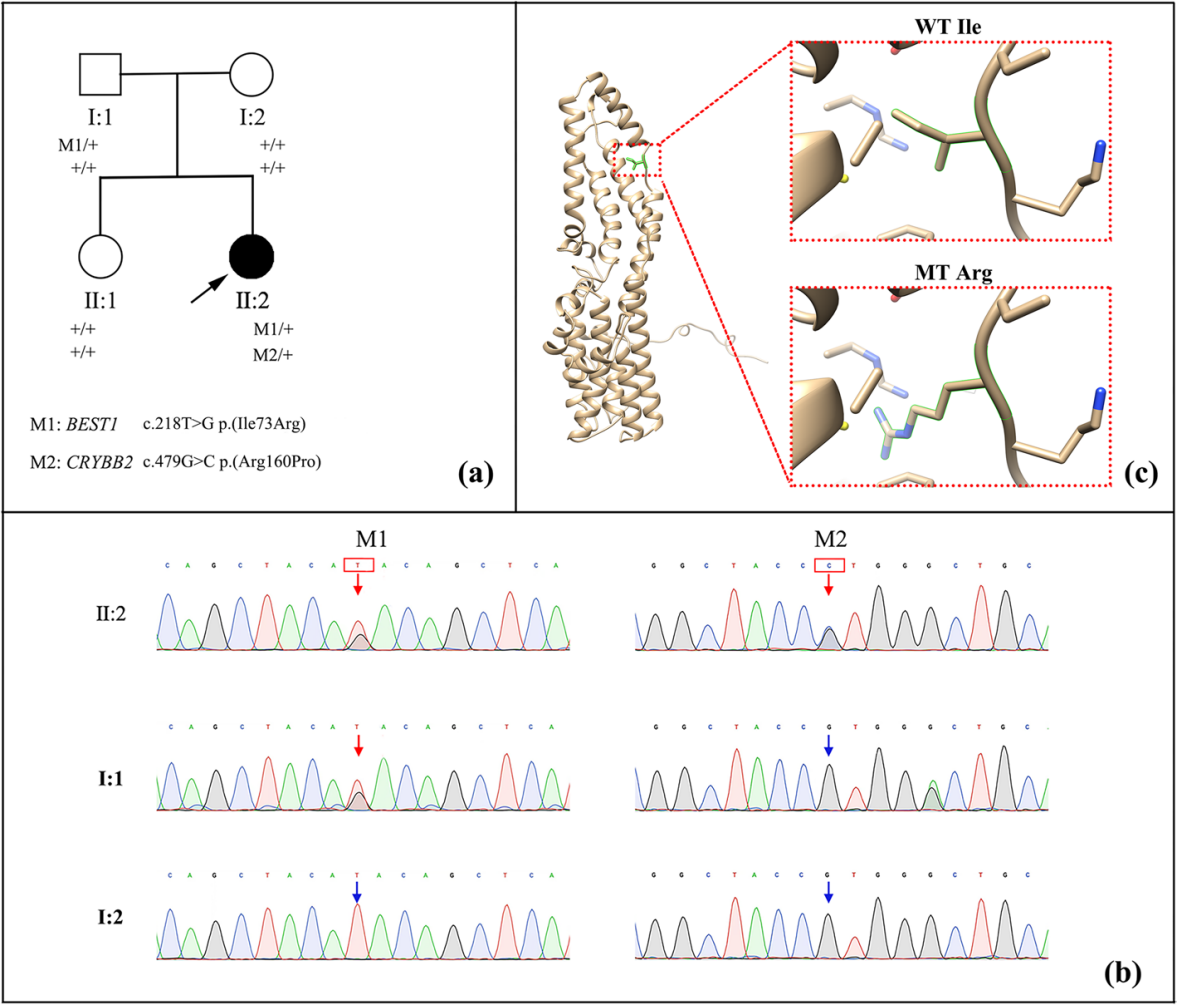
Genetic analysis of the family. **a** Pedigree of this family. **b** Sanger sequencing confirmed variants c.218 T > G p.(Ile73Arg) in *BEST1* and c.479G > C p.(Arg160Pro) in *CRYBB2*. The variant p.(Ile73Arg) in *BEST1* was inherited from the proband’s father, while p.(Arg160Pro) in *CRYBB2* was a de novo variant. The c.219C > A p.(Ile73 =) is a predominant (https://gnomad.broadinstitute.org/variant/11-61722645-C-A) major allele in Asia populations. Red arrows denote the mutant type. Blue arrows denote wildtype (WT). **c** Three-dimensional model of a bestrophin-1 subunit (the Protein Data Bank archive ID: 8D1I) and detailed structures of the WT and variant p.(Ile73Arg) proteins. The proteins were modeled in UCSC Chimera. MT, mutant type

The whole exome sequencing (WES) for the proband revealed two novel heterozygous missense variants: c.218T>G p.(Ile73Arg) in *BEST1* and c.479G>C p.(Arg160Pro) in *CRYBB2*. The details of WES were shown in Additional file 1. Cosegregation analysis showed that the variant p.(Ile73Arg) in *BEST1* was inherited from her father, while p.(Arg160Pro) in *CRYBB2* was a *de novo* variant (Fig 3a and b). The two variants were predicted to be pathogenic by at least two of three *in silico* analysis programs (Mutation Taster, PolyPhen2, and SIFT). Three-dimensional models showed distinct structures of the altered amino acid between wild type and mutant proteins of BEST1(Fig 3c). Neither variant was recorded in ExAC, 1000G or GnomAD. We classified c.218T>G in *BEST1* (PM1+PM2+PM5+PP2+PP3+PP4) and c.479G>C in *CRYBB2*(PS2+PM2+ PP3) as likely pathogenic according to the American College of Medical Genetics and Genomics (ACMG) criteria [9]. As several previously reported missense variants in *BEST1*are known to cause ADVIRC by affecting pre-mRNA splicing [2, 10, 11], we performed minigene assays to assess the impact of c.218T>G on splicing using a pET01-based exon trapping system (Exontrap; MoBiTec GmbH, Goettingen, Germany). An additional file shows this in more detail [see Additional file 1]. The minigene results showed that variant c.218T>G did not cause abnormal splicing [eFigure 1, see Additional file 1].

Based on the clinical and genetic findings, we diagnosed bilateral congenital cataract, microcornea, microphthalmia, and BVMD in the proband. Her father was diagnosed with BVMD (previtelliform stage).

## Discussion and conclusions

In this study, we described a patient suffering from bilateral microcornea, microphthalmia, congenital cataract, and BVMD caused by variants in *BSET1* and *CRYBB2*. The *BEST1* variants can cause both BVMD and ADVIRC. Patients with ADVIRC usually have microphthalmia, microcornea, and cataract, while patients with BVMD only show an egg-yolk lesion in the macula. Our case presented a special phenotype with overlapping features of both BVMD and ADVIRC.

The variant c.218 T > G p.(Ile73Arg) in *BEST1*is novel, but substitutions of isoleucine at residue 73 with asparagine, methionine, leucine, and valine have been reported in patients with BVMD [10–13]. Moreover, the proband’s father showed a mildly abnormal retinal structure and decreased Arden ratio, which implied only subclinical BVMD but no other ocular malformations. Previous studies have reported five variants p.(Gly83Asp), p.(Val86Met), p.(Val235Ala), p.(Tyr236Cys), and p.(Val239Met) identified in patients with ADVIRC. The variations were distributed around the neck of calcium-activated anion channels, and four of them (p.(Gly83Asp) was the exception) caused abnormal splicing according to minigene assays [2, 14, 15]. The novel variant identified in the current study was located outside the neck region [eFigure 2, see Additional file 1], and our minigene result showed that the novel variant did not affect *BEST1*pre-mRNA splicing. Although the overall globe parameters (corneal diameter and axial length) in the patient were close to ADVIRC phenotypes, but the cataract in ADVIRC was acquired but not congenital, moreover the retinal appearance was quite different. The proband and her father had no extensive choroidal or retinal atrophy with far-peripheral retinal circumferential hyperpigmented bands or fibrillar vitreous condensation, which are the retinal features of ADVIRC [2]. Taking these considerations together, we inferred that the p.(Ile73Arg) variant in *BEST1* was responsible only for the BVMD phenotype of the proband, while the CC, microcornea, and microphthalmia were caused by the *CRYBB2* variant.

The *CRYBB2*gene encodes βB2-crystallin which contains four Greek key motifs, encoded, separately by exons 3 to 6 [6]. At present, 42 disease-causing variants have been reported in the *CRYBB2*gene (HGMD Professional 2021.4), and most of them were missense variants located in Greek keys III (103-150AA) and IV (151-191AA) [7]. Several *CRYBB2*variants (p.(Arg145Trp), p.(Val146Met), p.(Gln147Arg), p.(Gly149Val), and p.(Thr150Met)) clustered in exon 5 have been reported to cause congenital cataract with other ocular abnormalities, such as microcornea, microphthalmia, ocular coloboma, and glaucoma [6, 8, 16]. It is unclear why these variants cause the combined phenotype, while the others only lead to cataract [6]. The novel variant p.(Arg160Pro) identified in the proband is the first one in exon 6 (Greek key IV) to be associated with microcornea and microphthalmia.

In this case, variants in two genes were identified in a patient with a complex ocular phenotype consisting of microphthalmia, microcornea, cataract, and vitelliform macular dystrophy. Comprehensive clinical and genetic assessments are crucial for precise diagnosis.

## Supplementary Information

**Additional file 1. **

**Additional file 2. **

## Data Availability

Not applicable.

## Abbreviations

BVMD: Best vitelliform macular dystrophy
WES: Whole exome sequencing
ADVIRC: Autosomal dominant vitreoretinochoroidopathy
CC: Congenital cataract
IOP: Intraocular pressure
OD: Right eye
OS: Left eye
BCVA: Best corrected visual acuity
SD-OCT: Spectral domain optical coherence tomography
FAF: Fundus autofluorescence
EOG: Electrooculogram
ACMG: The American College of Medical Genetics and Genomics
