# Supplementary material for: Variants of BEST1 and CRYBB2 cause a complex ocular phenotype comprising microphthalmia, microcornea, cataract, and vitelliform macular dystrophy: case report
Source: BMC Ophthalmol. 2023 Apr 19;23:165. doi: 10.1186/s12886-023-02915-3 (PMC10114320; doi:10.1186/s12886-023-02915-3)

**Additional file 1**

**eMethods**

**eFigure 1**

This supplementary material has been provided by the authors to give readers additional information about their work.

**eMethods**

**Whole exome sequencing (WES)**

Genomic DNA(1-3μg) was fragmented into 300-450 base pairs by endonuclease digestion, which were then utilized to capture the genomic exon sequences. The enrichment libraries were sequenced on platform NextSeq 500 (Illumina, Inc., San Diego, CA, USA) as 100-bp paired-end reads, according to the Manufacturer’s procedure. The high-quality reads were aligned with the reference human genome (hg19) by the Burrows–Wheeler Aligner after removing low-quality or duplicated reads using fastq_mcf software and Picard tools. Single nucleotide polymorphisms (SNPs) and small deletions or insertions were called using the Genomic Analysis Toolkit Haplotype Caller. These variants were further annotated using ANNOVAR.

**Minigene Assay in Human embryonic kidney-293T (HEK-293T) Cells**

The variant c.218T>G in *BEST1* was evaluated for an effect on splicing with the pET01-based exon trapping system (Exontrap, MoBiTec GmbH, Goettingen, Germany). Sequences including exon 3 (c.218T>G in exon3) and exon4 of *BEST1* were PCR amplified from genomic DNA using oligonucleotide primer pairs (Primer1 and 2). The 5’ end of the forward primers was designed to include a BamHI site (GGATCC), and the 5’ end of the reverse primers included a NotI site (GCGGCCGC). The amplified products were cloned into the pET01 vector. Clones with wild-type or mutant-type alleles were detected and screened by Sanger sequencing.

HEK293T cells were maintained in Dulbecco’s modified Eagle medium/High Glucose (Cytiva) supplemented with 10% fetal bovine serum (Sigma) and 1% Penicillin Streptomycin (Gibco). The cells were transfected with 3 µg of the selected minigene plasmids using Lipofectamine 2000 DNA transfection reagent (Invitrogen, Carlsbad, CA, USA). After 48 h, the transfected cells were harvested, and total RNA was extracted using the RNAprep Pure Cell/Bacteria kit (Tiangen, Beijing, China). The reverse transcription PCR (RT-PCR) was performed with a pair of specific primers ETPR04 and ETPR05 in the exons of pET01) and the FastKing One-Step RT-PCR Kit (Tiangen Biotech, China). The products were separated by electrophoresis on 2% agarose gels, excised, and sequenced.

Primer sequences:

Primer1 (Forward): 5’-GCGCGGATCCgtgtctctttgctcctcccc-3’;

Primer2 (Reverse): 5’-GCGCGGCCGCcgatcttcccacctttgcct-3’

Primer ETPR04: 5’-GGATTCTTCTACACACCC-3’

Primer ETPR05: 5’-TCCACCCAGCTCCAGTTG-3’

**eFigure 1.**

**Splicing results of minigene assays for the variant in *BEST1* in the HEK-293T cell line.** The variant c.218T>G did not show an abnormal splicing band. V1 and V2 are exons in pET01. M1, *BEST1* c.218T>G p.(Ile73Arg); WT, wildtype. The asterisk indicates the fragment without sequence information.

**
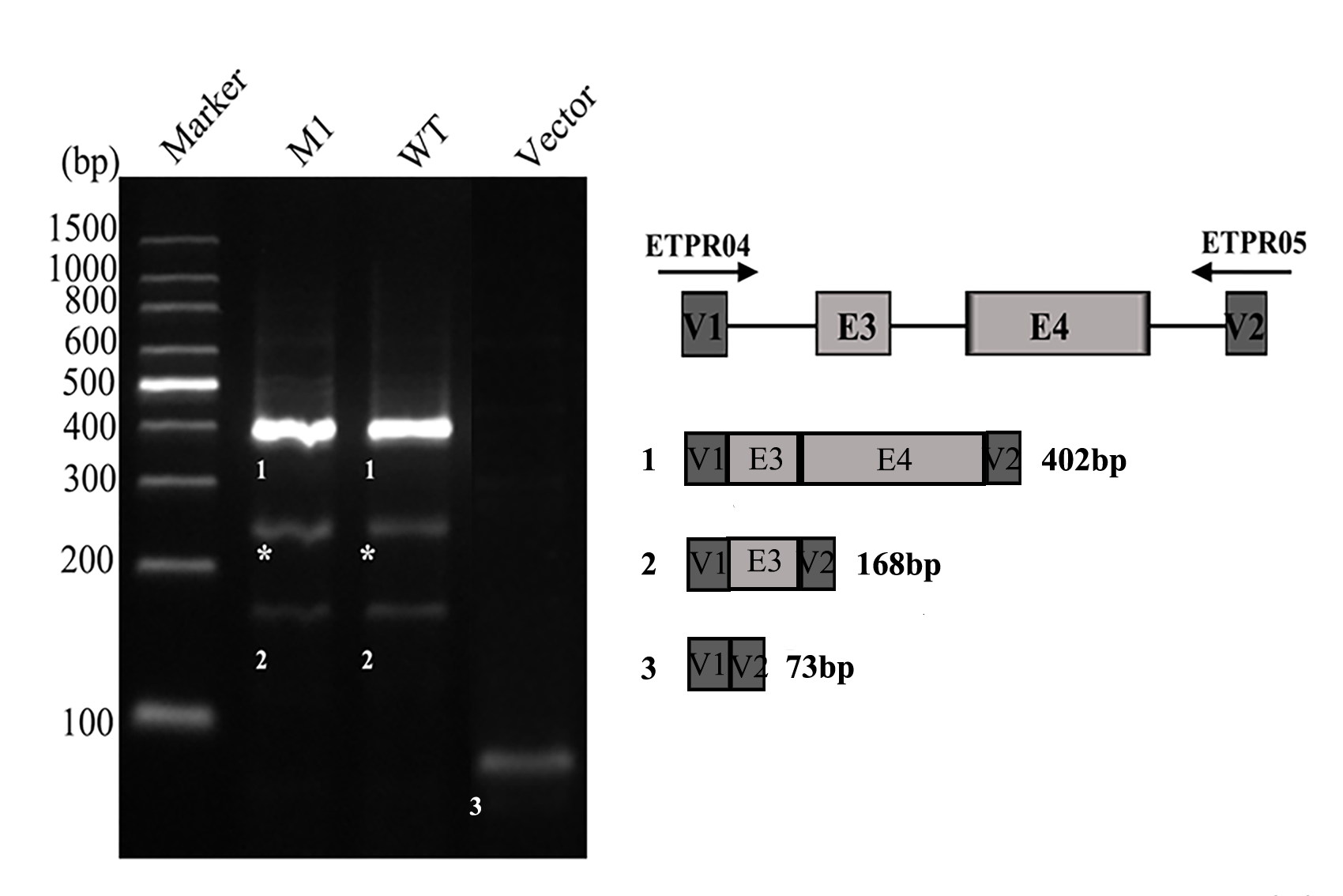
**

**eFigure 2.**

**Locations of variants in a 3-dimensional model of a bestrophin-1 subunit (the Protein Data Bank archive ID: 8D1I).** The five variants identified in patients with ADVIRC from other studies were noted as blue. The variant identified in our study was noted as red.


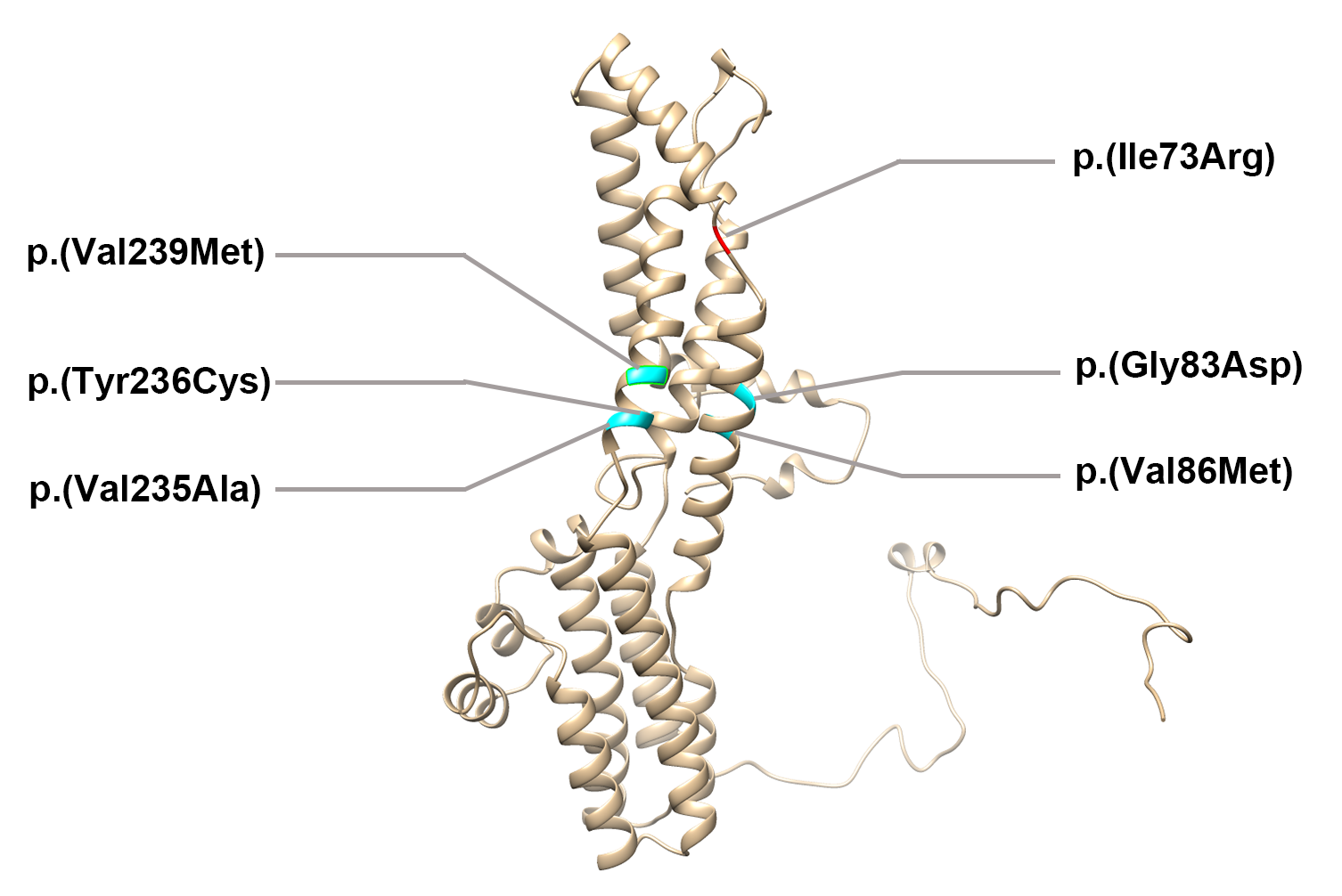

Supplement: Supplementary file 1 — Additional file 1. [file 12886_2023_2915_MOESM1_ESM.docx]
