# Supplementary material for: Variants of BEST1 and CRYBB2 cause a complex ocular phenotype comprising microphthalmia, microcornea, cataract, and vitelliform macular dystrophy: case report
Source: BMC Ophthalmol. 2023 Apr 19;23:165. doi: 10.1186/s12886-023-02915-3 (PMC10114320; doi:10.1186/s12886-023-02915-3)
Supplement: Supplementary file 2 — Additional file 2. [file 12886_2023_2915_MOESM2_ESM.docx]

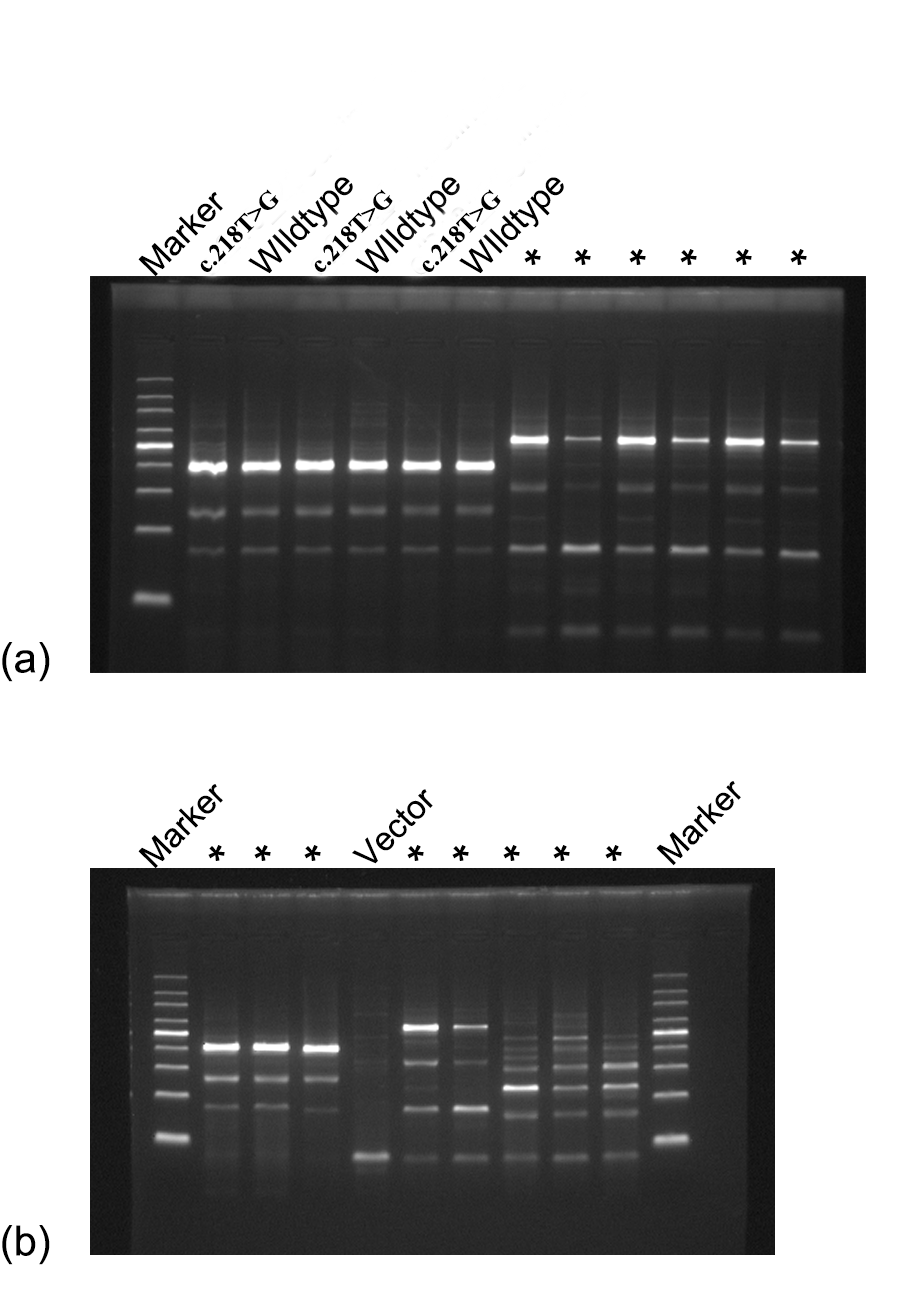


**Figure Legend:**

The original images of the gel electrophoresis in Figure 3.

1. Splicing results of minigene assays for the variant in *BEST1* in the HEK-293T cell line. The variant c.218T>G did not show an abnormal splicing band. (b) The vector band is shown in another gel electrophoresis image. The asterisk indicates electrophoretic bands not relevant to this study.
